# The United States Medical Licensing Examination Step 1 Is Changing—US Medical Curricula Should Too

**DOI:** 10.2196/20182

**Published:** 2020-07-30

**Authors:** Benjamin Liu

**Affiliations:** 1 Medical College of Wisconsin Wawautosa, WI United States

**Keywords:** USMLE, US medical students, USMLE pass/fail, new curricula, medical education, medical learning, medical school

## Abstract

In recent years, US medical students have been increasingly absent from medical school classrooms. They do so to maximize their competitiveness for a good residency program, by achieving high scores on the United States Medical Licensing Examination (USMLE) Step 1. As a US medical student, I know that most of these class-skipping students are utilizing external learning resources, which are perceived to be more efficient than traditional lectures. Now that the USMLE Step 1 is adopting a pass/fail grading system, it may be tempting to expect students to return to traditional basic science lectures. Unfortunately, my experiences tell me this will not happen. Instead, US medical schools must adapt their curricula. These new curricula should focus on clinical decision making, team-based learning, and new medical decision technologies, while leveraging the validated ability of these external resources to teach the basic sciences. In doing so, faculty will not only increase student engagement but also modernize the curricula to meet new standards on effective medical learning.

For US medical students, 2020 has been a wild year. In addition to the pandemic and an increasing number of zero tuition medical schools, the United States Medical Licensing Examination (USMLE) Step 1 has adopted a pass/fail grading system. The percentile students scored on this test has historically been one of the most important determinants of which residency program or even which area of medicine students could apply to. Now that it is pass/fail, doing anything more than just passing has no effect on a student’s residency competitiveness. While I agree with most of the reasons for this change, the glaring question remains: what will the medical school curriculum look like now that Step 1 has become pass/fail? As a second-year medical student attending a typical mid-west US medical school, I believe I can offer a first-hand perspective on student engagement with the traditional, lecture-based medical curriculum, and the major changes that should occur as students shift their focus away from scoring a 230+ on Step 1.

Historically, US medical schools have had a heavy emphasis on memorization, a matter exemplified by the 2 years of traditional lectures dedicated for the basic science–heavy Step 1. However, in the modern era, we acknowledge that medical knowledge has proliferated to the point where it is impossible to memorize everything. Nevertheless, the heavy emphasis on Step 1 scores for residency admissions leads medical students to devote significant time and energy to maximize their scores. According to the 2019 Medical School Year Two Questionnaire administered by the Association of American Medical Colleges, US medical students have been largely absent in traditional, lecture-based classes, and over one-third (34.9%) of medical students “never” or “occasionally” attend virtual classes [1]. Instead, an increasing number of students—including myself—pursue a “parallel curriculum” of dedicated Step 1 preparation [1-3]. This involves external medical resources (eg, Pathoma, boards, etc) that feature short and concise lectures (playable at double speed), with accompanying spaced repetition “Anki” flashcards. These resources have provided a basic medical science learning platform that students are flocking to instead of traditional lectures [1-3]. Unfortunately, this has resulted in empty medical classrooms, faculty frustration with students, and decreased faculty enthusiasm for teaching [4].

Since the driving force for these behaviors are Step 1 mediated, you may wonder if students will re-engage with traditional basic science lecture formats now that Step 1 is pass/fail. I believe the answer will be no. Even without the pressures of Step 1, students will not attend if they feel they are not efficiently using their time. Many articles have been published showing that the amount of content covered in traditional medical lectures overwhelms the brain's ability to learn [5]. As for the information that does get across, students often only understand it at a superficial level [5]. Given that students still need to choose between doing research, extracurriculars, and learning the basic science to pass Step 1, students will always pick a 10-15–minute video at double speed over a 1-hour in-person lecture [1,3,6]. To remain relevant, I believe that the current medical curriculum must undergo significant re-evaluation and transformation. With this change, faculty should leverage external resources to help their in-person classes focus on teaching the loftier aspects of medicine: clinical decision making, effective utilization of technologies, and most importantly, working effectively in a team-based environment.

In the past, both faculty and students have focused less on clinical decision making and interprofessional education in favor of Step 1 performance, resulting in students with limited practical skills before clerkship. With Step 1 becoming pass/fail, future curricula have an opportunity to fully embrace teaching these skills through a flipped classroom model. Schools have already recognized the value of the flipped classroom model, and several have implemented them on a consistent basis. This relatively new classroom style involves students learning the material beforehand, as directed by assigned readings or video modules, and then applying it in classroom-based clinical scenarios. It addresses student's preferences for self-paced basic science learning, teaches critical thinking and team-based collaboration, and leads to improved outcomes for medical student learning compared to traditional lectures [7]. As described by one perspective published in the *New England Journal of Medicine*, flipped classrooms allow concepts to be “stickier” by making a student actively apply the content to a relevant and interesting scenario soon after learning it [8]. This reinforces the value of the knowledge, making it more understandable and memorable than passively learning a dry and complex biochemical pathway for an hour [5,8]. Moreover, as one of my preceptors told me, “real medicine is not a test. It involves gathering information from everyone in the room and coming up with a list of the best possible explanations before investigating each one.” In hindsight, a well-designed collaborative flipped classroom appears to be the exact formula for real medicine.

Finally, the historical focus on rote memorization is not well adapted to today’s age where all minute details can be discovered with the click of a button. As a second-year student, I would have appreciated formal training in utilizing current medical technologies to supplement our memory. Now that students will no longer be pressured by Step 1 into memorizing everything, future curricula can go beyond simply mentioning clinical decision-making tools (CDMTs) such as UpToDate and ASCVD Risk Estimator Plus. Students should be consistently taught to engage with these technologies via clinical cases. This will lead to familiarity, and therefore more effective use of these technologies, which have already been proven to lead to better patient outcomes when used appropriately [9]. Curricula that incorporate effective utilization of CDMTs can also help eliminate the stigma against using them in clinical practice [9,10]. From the patient's perspective, this stigma is unfounded [9,10]. Effective use of CDMTs is appreciated by patients—who increasingly understand that physicians cannot be expected to memorize every little detail—so long as the physician communicates what they are doing. For physicians, this stigma comes from the perceived interference these technologies have on their face-to-face interactions with the patient, as well as a perception that reliance on such technologies suggests decreased medical ability. While the latter point likely has its roots in metrics that overly prioritized memorization (eg, Step 1), the first point is a very valid point against electronic health records. However, CDMTs are different and can be an opportunity to build rapport with patients by demonstrating engagement with the patient's concern as well as providing trusted resources for the patients to read more about. Therefore, training future physicians to be comfortable with CDMTs, with the caveat of also training them to effectively incorporate CDMTs in their face-to-face interactions (eg, turning the screen toward the patient), is a critical change that medical curricula must make.

In conclusion, schools should not fall back on traditional, lecture-based curricula in response to the Step 1 changes. They must modernize their curricula by promoting active, team-based learning that incorporates the many technologies that have and will continue to revolutionize the field of medicine. After all, we change our medical practices based on new evidence and guidelines. We should do the same for medical education.

## Abbreviations

CDMT: clinical decision-making tool
USMLE: United States Medical Licensing Examination
